# The Impact of Pancreatic Exocrine Diseases on the β-Cell and Glucose Metabolism—A Review with Currently Available Evidence

**DOI:** 10.3390/biom12050618

**Published:** 2022-04-21

**Authors:** Marina Ciochina, Daniel Vasile Balaban, George Manucu, Mariana Jinga, Cristian Gheorghe

**Affiliations:** 1 Faculty of Medicine, Carol Davila University of Medicine and Pharmacy, 050474 Bucharest, Romania; vasile.balaban@umfcd.ro (D.V.B.); mariana.jinga@umfcd.ro (M.J.); cristian.gheorghe@umfcd.ro (C.G.); 2 Gastroenterology Department, Central Military Emergency University Hospital, 010825 Bucharest, Romania; george.manucu@rez.umfcd.ro; 3 Gastroenterology Department, Fundeni Clinical Institute, 022328 Bucharest, Romania

**Keywords:** pancreatic β-cells, diabetes mellitus, pancreatic exocrine insufficiency, chronic pancreatitis, acute pancreatitis, cystic fibrosis, pancreatic surgery, pancreatic adenocarcinoma, autoimmune pancreatitis

## Abstract

Pancreatic exocrine and endocrine dysfunctions often come together in the course of pancreatic diseases as interdependent manifestations of the same organ. However, the mechanisms underlying the bidirectional connection of the exocrine and endocrine pancreas are not fully understood. In this review, we aimed to synthetize the current knowledge regarding the effects of several exocrine pancreatic pathologies on the homeostasis of β-cells, with a special interest in the predisposition toward diabetes mellitus (DM). We focused on the following pancreatic exocrine diseases: chronic pancreatitis, acute pancreatitis, cystic fibrosis, pancreatic cancer, pancreatic resections, and autoimmune pancreatitis. We discuss the pathophysiologic mechanisms behind the impact on β-cell function and evolution into DM, as well as the associated risk factors in progression to DM, and we describe the most relevant and statistically significant findings in the literature. An early and correct diagnosis of DM in the setting of pancreatic exocrine disorders is of paramount importance for anticipating the disease’s course and its therapeutical needs.

## 1. Introduction

The pancreas is well known for its dual role: the exocrine role, which is responsible for the secretion of digestive enzymes, and the endocrine role, which is engaged in the secretion of pancreatic hormones. These roles are no longer seen separately, but as two interdependent functions of the same organ.

Distinct from the two main types of diabetes mellitus (DM), type 1 diabetes mellitus (T1DM) (described by the presence of autoantibodies against insulin-producing pancreatic β-cells and the absence of insulin secretion) and type 2 diabetes mellitus (T2DM) (with peripheral insulin resistance, no autoantibodies, and impaired insulin secretion), a new category was defined, type 3c diabetes (T3cDM), or pancreatogenic or pancreoprivic diabetes mellitus, which has been reported to account for 9% of patients hospitalized with diabetes [1,2]. 

T3cDM, also called diabetes of the exocrine pancreas (DEP), includes perturbations of the glycemic metabolism that are secondary to exocrine pancreatic disorders. Among them, chronic pancreatitis appears to be responsible for approximately three-quarters of T3cDM [1]. Others have reported that acute pancreatitis could account for around 60% of DM associated with pancreatic exocrine disease, but glycemic abnormalities in this setting might be transient [3].

T3cDM is often not correctly recognized. Approximately 40% of hospital inpatients with diabetes of the exocrine pancreas are misdiagnosed as T2DM [1]. 

In T3cDM, the damage is not limited to pancreatic β-cells, as it is in T1DM, but to all cell subtypes within the islet of Langerhans. This leads to a global secretory deficiency that includes insulin, glucagon, pancreatic polypeptide, and somatostatin (Table 1). The correct diagnosis of pancreatogenic diabetes is vital, as, in contrast with T2DM, it is more frequently associated with poor glycemic control, inducing the so-called “brittle diabetes”, and the need for insulin therapy is often present earlier in the disease’s course. A study by Woodmansey et al. [4] revealed that insulin use within 5 years of the diagnosis of DM was significantly higher in T3cDM (20.9% with DM after acute pancreatitis and up to 45.8% with diabetes following chronic pancreatitis) than in T2DM (4.1%).

**Figure 1:**
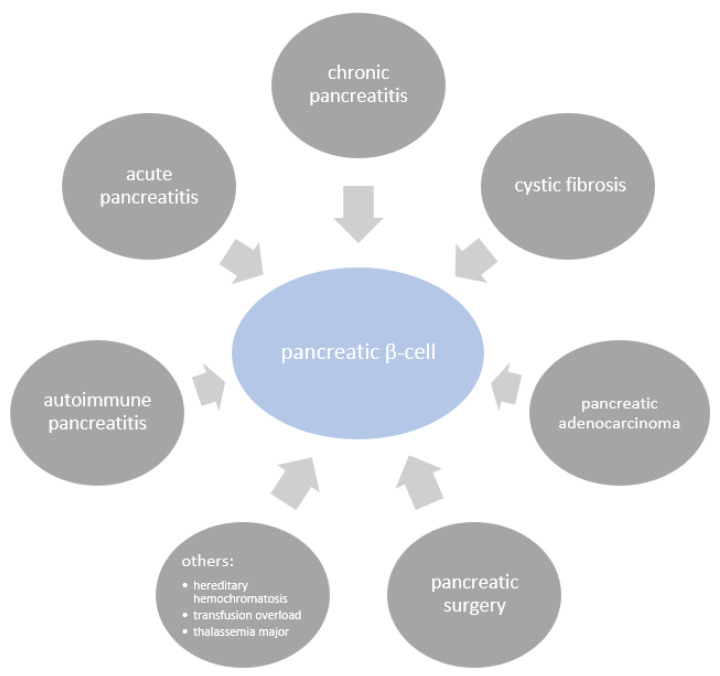
The most common pancreatic exocrine diseases responsible for T3cDM.

Chronic and acute pancreatitis are the most common causes of T3cDM. Additionally, there are other diseases of the exocrine pancreas involved in glucose metabolism perturbations (Figure 1). In this article, we reviewed relevant papers related to cystic fibrosis, pancreatic adenocarcinoma, pancreatic surgical resections, and autoimmune pancreatitis, along with those referring to acute and chronic pancreatitis (CP). Conditions such as hereditary hemochromatosis, transfusion overload, and thalassemia major are commonly responsible for diabetes of the exocrine pancreas, but they are not discussed in this article, as they are rather systemic conditions and not organ-centered pathologies. However, patients with these conditions should also be screened for T3cDM [5].

## 2. Search Strategy

For the purpose of this review, we conducted a literature search for published manuscripts on pancreatic exocrine pathologies and their implications for β-cell and glucose metabolism. The search was performed in January 2022 on the PubMed (MEDLINE) database, using the following Mesh terms: “Insulin-Secreting Cells” (ID D050417), “Pancreatic Diseases” (ID D010182), and “Glucose Metabolism Disorders” (ID D044882). The search was restricted to full-text English-language articles and excluded case reports and case series, narrative reviews, letters, and papers referring to animal studies. 

We screened titles according to relevance to the topic researched. Among the pancreatic exocrine pathologies, we included chronic pancreatitis, acute pancreatitis, cystic fibrosis, pancreatic cancer, pancreatic resections, and autoimmune pancreatitis. For the pancreatic masses, we excluded papers related to neuroendocrine tumors, as their secretory properties could alter the interpretation of pancreatic function. Finally, a total of 60 papers were selected for analysis. 

## 3. Chronic Pancreatitis

One of the most common pancreatic exocrine pathologies is chronic pancreatitis (CP). The most important pathway for progression to DM is inflammation with its local and systemic consequences, together with β-cell dedifferentiation and, finally, β-cell loss.

### 3.1. Risk Factors

The relationship between CP and impaired β-cell function was discussed in several studies. A multicenter study of 1117 cases conducted by Olesen at al. [7], aiming to analyze the risk factors for developing DM in patients with CP, found that general risk factors for DM, such as dyslipidemia, overweight, and obesity, were associated with increased prevalence of DM in CP patients as well. Other significant and independent risk factors for DM in CP patients were age at diagnosis, duration of CP, pancreatic calcifications, pancreatic resection, and pancreatic exocrine insufficiency (PEI) [7].

### 3.2. Timing

DM is a delayed complication of long-standing CP, with approximately 50% of patients having DM 10 years after a diagnosis of CP and 83% at 25 years after diagnosis according to Malka et al. [8]. Lower percentages were found by Wang et al. in a study on a Chinese population, with a cumulative rate of 24% at 10 years and of 52% at 20 years of CP [9]. A similarly high prevalence of DM was found by Pan et al. in a cohort of 2011 patients; they reported that 90% of patients had diabetes of the exocrine pancreas after 50 years of CP evolution [10].

In contrast, recent studies found that DM is not restricted to patients who have had CP for decades, revealing that DM can develop earlier in the course of CP. In a study by Olesen at al., 61% of patients developed DM less than 5 years after the diagnosis of CP [7].

### 3.3. Mechanism

The most relevant underlying mechanism of increased risk of DM is the gradual β-cell loss due to the evolving inflammatory process. Additionally, it is believed that the genetic predisposition for DM also plays a key role in the progression of CP to DM [7].

Among the mechanisms of DM in CP patients, there is also the dedifferentiation of pancreatic β-cells. Under certain metabolic stress conditions, such as oxidative stress, endoplasmic reticulum (ER) stress, inflammation, hypoxia, or even glucotoxicity induced by hyperglycemia, mature β-cells lose their phenotype and regress to a precursor-like dedifferentiated state [11]. Through dedifferentiation, the β-cells lose the genes that are responsible for their identity and maturity and acquire a new progenitor-like phenotype with a different potency [12]. 

The inflammation of the pancreatic parenchyma is considered the trigger for β-cell dedifferentiation and dysfunction, according to in vitro studies [8,13]. The key mechanism responsible for the high risk of DM in CP is the decrease in insulin production and the reduced expression of PDX-1 (insulin promoter factor 1)—a β-cell-specific transcription factor whose expression is reduced by inflammation [14,15].

A significant increase in dedifferentiated cells was also seen in vivo in non-diabetic CP patients compared with normal subjects, with most of the dedifferentiated cells being located in the so-called nonhormone empty regions of the islet. The degree of dedifferentiation was correlated with the severity of the inflammation, fibrosis, or atrophy of the pancreas [11]. 

As previously mentioned, inflammation is the most important cause of β-cell damage and loss during the progression of CP. Regarding the exact pathways of T cell-mediated inflammation, a study conducted by Talukdar et al. [16] found an elevation in the circulation of T-helper 1 lymphocytes (Th1) and T-helper 17 lymphocytes (Th17) in CP patients without diabetes and a decrease in Th17 cells in CP patients with diabetes. This suggests the plasticity of Th17 cells, which, under an inflammatory environment, differentiate into Th1 cells. No significant alterations in T-helper 2 lymphocytes (Th2) or regulatory T cells lymphocytes (Treg) were noted [16]. When analyzing the local intrapancreatic inflammation, an increased number of both Th1 and Th17 cells was described, leading to an elevated intrapancreatic interferon γ (IFN-γ) level, which is responsible for alterations in the signaling mechanisms of insulin gene transcription, thereby leading to β-cell dysfunction early in the course of the disease [16,17]. 

The role of IFN-γ in pancreatic inflammation is essential due to its deleterious character in the signaling mechanism of insulin gene transcription. Elevated intrapancreatic IFN-γ concentration leads to JAK-STAT (Janus kinase/signal transducer and activator of transcription) stimulation. STAT1 is an important transcription factor of the IFN-γ signaling mechanism with a crucial role in pancreatic inflammation. It is also important in reducing insulin gene transcription by exporting phosphorylated PDX-1 from the nucleus into the cytoplasm. A 12-fold increase of STAT1 was observed in pancreatic tissue, together with a decrease in phosphorylated PDX-1 [16]. 

Even if β-cell dysfunction can appear early in the course of CP, DM is usually a late complication. Mitnala et al. showed an islet reduction of 47% before clinical diabetes was evident [14]. Alterations in the internal environment of pancreatic parenchyma determine qualitative and quantitative changes in cytokine signaling pathways, which are responsible for damage to β-cells [18]. Aside from their role in generating β-cell apoptosis as a normal adaptive response, some of the proinflammatory cytokines, such as interleukin 1b (IL-1b), tumor necrosis factor α (TNF-a), and IFN-γ, play a role in inhibiting the hormone secretion activity [19]. Insulin-like growth factor-1 (IGF-1) and gastrointestinal hormones, together with cytokines and chemokines, have a significant role in influencing the islet function. In addition, the role of cytokines in β-cells is dose-dependent, stimulating insulin synthesis and secretion at low concentrations, while causing progressive functional impairment and β-cell death at higher concentrations [18,20]. Low concentrations of IL-1b stimulate β-cell proliferation, while higher concentrations lead to functional impairment and β-cell loss through necrosis and cell apoptosis. The apoptosis induced by IL-1b and IFN-γ may also be the result of activation of nuclear factor κB (NF-κB) and mitogen-activated protein kinases (MAP kinases), such as p38 mitogen-activated protein kinases and extracellular signal-regulated kinases (ERK) [18]. Furthermore, cytokines influence the β-cells’ capacity to produce ATP by reducing cAMP synthesis. They inhibit the glucagon-like peptide-1 (GLP-1) and gastric inhibitory peptide receptors and, thereby, determine the reduction of the cAMP formation that leads to a decreased β-cell survival [21].

## 4. Acute Pancreatitis

Acute pancreatitis (AP) is one of the most frequent diseases of the pancreas. A large epidemiological study by Woodmansey et al. found that AP is the most common pancreatic disease responsible for the development of diabetes [4]. 

A population-based cohort study in Taiwan in 2016 [22] described the bidirectional relationship between AP and DM. It revealed that the adjusted hazard ratio of AP was significantly higher in patients with DM, with higher values in those with a history of hyperglycemic crisis episodes, including ketoacidosis and a hyperosmolar hyperglycemic state. Furthermore, the adjusted hazard ratio of DM was increased in patients with AP compared to the general population, without significant differences in those with severe episodes of AP compared with non-severe AP [22].

### 4.1. Risk Factors

Regarding the risk factors for the development of impaired β-cell function after AP, the same meta-analysis by Das et al. [23] also revealed that the severity of the AP episode, etiology, age, and gender did not play a significant role in the development of prediabetes or DM [23]. The body mass index was not analyzed in this meta-analysis.

Some articles that were published later found different results. A large nationwide population study in New Zealand reported that men have a 40% higher risk of developing diabetes of the exocrine pancreas than women. Moreover, acute pancreatitis was responsible for 61% of the overall prevalence of diabetes in patients with pancreatic exocrine diseases [3]. 

Additionally, a study by Tu J et al. [24] that included 256 patients with AP revealed that pancreatic necrosis and persistent organ failure were significant risk factors for developing new-onset DM (NOD). Higher prevalence of T3cDM was also described in severe episodes of AP by Vujasinovic et al. in a study that found an incidence of 14% of T3cDM after AP [25].

In order to identify patients with a higher risk of impaired glucose metabolism after AP, a new score called PERSEUS (Prediabetes self-assEssment scReening Score after acUte pancreatitiS) was developed. It has the advantage that it does not require a laboratory work-up, as it is based on self-reported factors, allowing the patients to self-assess and monitor their own risk of developing prediabetes after hospital discharge [26].

### 4.2. Timing

A systematic review and meta-analysis by Das et al. [23], which included 24 prospective clinical studies and 1102 patients, showed that the pooled prevalence of prediabetes, DM, and insulin treatment was 16%, 23%, and 15%, respectively, in patients with a first episode of AP. Furthermore, 15% of patients developed DM within a year after the first episode of AP, with a risk of DM that was increased greater than twofold at 5 years (40%) [23]. 

### 4.3. Mechanism

The most relevant mechanism of DM after AP is considered the loss of pancreatic β-cells due to pancreatic necrosis. Extensive pancreatic necrosis determines the loss of functional pancreatic tissue and reduction of β-cells [24]. However, there are authors who deny this hypothesis, as in their studies, there were no relationships between the severity of AP and the development of DM [23]. An explanation could be that AP triggers an immune response in genetically susceptible patients for developing DM [23]. Another dominant role in developing T3cDM after AP could be the insulin resistance [24].

Among the AP severity criteria, the presence of organ failure and, mainly, the association between organ failure and pancreatic necrosis were found to be correlated with impaired pancreatic secretion, and especially with pancreatic exocrine insufficiency [24]. If β-cell loss is the obvious cause of DM after severe necrotizing AP, the pathogenesis of DM in mild AP is more complex. The DORADO project aimed to explain the pathophysiology of NOD after AP. Among the immune pathways, IL-6 was found to be the major cytokine involved in NOD after AP, causing an impaired phosphorylation of the insulin receptor and insulin receptor substrate-1 and leading to insulin resistance (a 1 ng/mL increase in IL-6 was associated with a 0.7% increase in insulin resistance) [27]. Thereby, increased levels of circulating insulin can be noted in DM after AP, including not only fasting levels, but post-prandial levels as well [28]. Another underlying mechanism of DM after AP is lipolysis through increased levels of glycerol and triglycerides. In addition, an increased circulating level of glycerol was associated with increased IL-6 and β-hydroxybutyrate [27].

In the DORADO project, one of the aims was to establish the gut peptides involved in NOD after AP. The results were in favor of a significant decrease in circulating levels of oxyntomodulin and glicerin, two proglucagon-derived peptides, as well as gastrin-releasing peptide (GRP), vasoactive intestinal peptide (VIP), and calcitonin gene-related peptide (CGRP). Oxyntomodulin levels were significantly associated with cholecystokinin and VIP, with decreased levels of oxyntomodulin in patients with DM after AP being accompanied by decreased levels of cholecystokinin and VIP as a reflection of exocrine pancreatic dysfunction. These data suggest that oxyntomodulin could be a biomarker for differentiating DM after AP from T2DM [27].

Finally, the higher risk of AP in patients with DM could be explained by the increased production of reactive oxygen species and increased lipid peroxidation in chronic hyperglycemia [29,30]. Furthermore, alterations in calcium metabolism, such as perturbed ryanodine receptor function, could be a way through which DM contributes to the development of AP [31].

## 5. Cystic Fibrosis

Cystic fibrosis (CF), an autosomal recessive disease, is a multisystemic disorder caused by abnormal chloride and bicarbonate transport related to mutations of the CFTR (CF transmembrane conductance regulator) protein, which leads to thick secretions in the lung, pancreas, liver, intestine, and reproductive tract.

Aside from the well-known exocrine manifestations, CF may be responsible for alterations in the pancreatic endocrine function and glucose metabolism. CF-related diabetes (CFRD) affects approximately 35% of adults with CF [32]. It is considered a form of T3cDM, but is often recognized as a distinct item, as it has features of both T1DM and T2DM.

### 5.1. Risk Factors and Timing

Dyslipidemia, which is normally associated with the risk of DM, was not found to be correlated with a higher susceptibility to hyperglycemia or CFRD [33]. In a comparison among CFRD, T1DM, and T2DM, Konrad et al. [34] revealed that the median age at diagnosis of CFRD (18.7 years) was between those of T1DM (16.4 years) and T2DM (58.5 years). Significant differences were noted regarding the body mass index (median of 19.6 in CFRD vs. 24.4 in T1DM vs. 29.6 in T2DM) and HbA1c level (median value 6.73% in CFRD vs. 7.83% in T1DM vs. 7.06% in T2DM), as well as the lower hypertension frequency in CFRD compared to T1DM and T2DM [34]. When comparing the health-related quality of life (HRQoL) in CFRD and T1DM, patients with CFRD had a less negative impact of diabetes on HRQoL, with fewer episodes of loss of consciousness and symptomatic hypoglycemia [35].

### 5.2. Mechanism

#### 5.2.1. β-Cell Loss

Regarding the mechanisms involved in the occurrence of DM, the majority of authors agree about β-cell loss and intra-islet inflammation with amyloid deposition. Patients with CFRD have a decreased density of islet tissue compared with CF patients without diabetes [32,36,37,38]. Interestingly, studies report the loss of β-cells with no changes in α-cells (glucagon-producing cells) or δ-cells (somatostatin-producing cells) [36,38].

The etiology of the β-cell mass loss in CFRD is multifactorial. Firstly, auto-digestion of the pancreatic tissue occurs as a result of the premature activation of the pro-enzymes, whose retention in the pancreatic ducts is favored by the pancreatic exocrine insufficiency. Then, the oxidative stress of β-cells and the malabsorption of dietary antioxidants in CF subjects, together with the misfolded CFTR proteins in the ER of the β-cells, can induce ER stress and β-cell apoptosis [37]. 

Another explanation could be the greater vulnerability of β cells to injuries, including hyperglycemia [38]. The counter-regulatory hormones, such as growth hormone, cortisol, and catecholamines, may temporarily alter the glycemic status. During puberty, growth hormone determines physiological insulin resistance and a greater rate of CFRD compared to younger children [38]. 

Finally, another mechanism of β-cell loss could be the ischemic damage induced by the reduced blood flow within the pancreatic tissue in the context of protein precipitations and ductal obstruction as a result of reduced chloride and bicarbonate ion secretion [39].

#### 5.2.2. Immunological Response

In the pathogenesis of CFRD, the immune cells’ infiltration of the islets is responsible for an inflammatory response with cytokine attraction and impaired β-cell function [32]. Hull, R.L. et al. performed a histological study, revealing a greater IL-1b immunoreactivity in all patients with CF, independently of the presence of the diabetes, but islet amyloid deposition only in patients with CFRD [36]. 

As for the systemic immune response, a hypothesis is that the fluctuations in the glycemic levels, especially hyperglycemia, together with low levels of vitamin D, may lead to lymphocyte dysfunction, with a disbalance between the pro-inflammatory Th17 lymphocytes and the anti-inflammatory Tregs lymphocytes. Th17 cells and dysfunctional Treg lymphocytes producing IL-17 seem to favor pulmonary inflammation and β-cell dysfunction [37].

#### 5.2.3. CFTR Expression

Another responsible mechanism could be the CFTR expression in pancreatic endocrine cells. Hart et al. denies the intrinsic role of CFTR in the regulation of α- or β-cell function, explaining the reduced secretory capacity mainly through the reduction in pancreatic β-cell areas in CF by 65% [32]. On the other hand, a small pilot study by Bellin et al. revealed that the CFTR is directly implicated in insulin secretion, and the correction of CFTR function could be the key to the improvement of glucose tolerance in CF patients [40]. The abnormal expression of CFTR in intralobular duct cells and pancreatic centroacinar cells remains a widely accepted hypothesis, as it is responsible for a reduction in chloride and bicarbonate ion secretion, causing reduced fluid secretion into pancreaticobiliary ducts and leading to protein precipitation and ductal obstruction [39].

Recently, some authors nominated the accumulation of misfolded CFTR proteins in the ER of the β-cells as a determinant of β-cell apoptosis [37].

#### 5.2.4. Impaired Insulin Secretion 

The reduction of β-cell areas drives reduced insulin secretion. Studies showed that the indicator of β-cell damage and progression to CFRD is the impairment of the first phase of insulin secretion after an oral or intravenous glucose overload. It can occur in subjects with CF and normal glycemic tests [41]. 

Another mechanism responsible for the impaired insulin secretion is the alteration of the entero-insular axis. Hormones implicated in insulin regulation, such as cholecystokinin, GLP-1, and glucose-dependent insulinotropic peptide, have an impaired secretion in CF patients [37]. 

However, in aging CF patients, the main role in the evolution into CFRD is played by decreased insulin sensitivity. In a retro-prospective observational analysis, Colomba et al. [42] showed that in patients with CF aged more than 35 years, the insulin secretion capacity was unchanged, while insulin sensitivity was reduced [42]. Furthermore, insulin sensitivity varies according to variations in glucose tolerance in adults with CF and patients without changes in glucose tolerance that have a stable insulin sensitivity, while those with impaired glucose tolerance have a worsening in insulin sensitivity [43]. 

#### 5.2.5. The Genotype

A study by Street et al. concluded that the genotype is a determinant factor for CFRD, while the age and the general condition of the patients do not have a significant role in inducing impaired glucose tolerance (IGT) and CFRD. Therefore, patients with a homozygote for the F508del, the most common CF mutation, were more susceptible to developing IGT and CFRD, while those with a heterozygote for the F508del mutation had a low prevalence of IGT and diabetes [44].

## 6. Pancreatic Surgery

Pancreatic surgery is a major determinant of the endocrine and exocrine function of the pancreas. There are debates regarding the importance of the extension of resection, the anatomical segment resected, and the surgical technique. The islet cells are interdependent, but their plasticity is not yet totally understood. The possibility of transdifferentiation of α-cells and δ-cells into insulin-containing β (like)-cells could be a novel strategy in the management of DM [45].

### 6.1. The Surgical Procedure

The part of the resected pancreas plays an important role, as pancreatic endocrine cells are distributed non-uniformly along the gland, with α-cells mostly in the tail, PP cells in the head, and β-cells throughout the entire pancreas [46]. Regarding the incidence of DM correlated with the type of pancreatic resection, the data are discordant. In a recent study by Jun Ishida et al., it was found that glucose tolerance and insulin sensibility are better after pancreaticoduodenectomy compared to distal pancreatectomy [47]. However, when performed for paraduodenal pancreatitis, pancreatoduodenectomy was correlated with a significantly higher incidence of diabetes compared to medical treatment, while the incidence of steatorrhea was similar between the two groups [48]. Furthermore, King et al. found a minimal (9%) rate of NOD after distal pancreatectomy, with an even lower rate (7.5%) between those without signs of pancreatitis [49].

A systematic review and meta-analysis by Beger et al. [50] showed an increased risk of postoperative NOD and pancreatic exocrine insufficiency in patients with pancreatoduodenectomy when compared to duodenum-preserving pancreatic head resection for benign tumors (NOD in 15% of patients with pancreatoduodenectomy vs. 6% in patients with preserved duodenum in the meta-analysis). The mean follow-up time was 20.7–22.2 months after surgery. These results are explained by the fact that duodenectomy is associated with postoperative impairment of gastrointestinal hormones, such as gastrin, secretin, cholecystokinin, insulin, PP, and gastric inhibitory polypeptide. Therefore, the reduction in pancreatic endocrine and exocrine secretion is more of a consequence of duodenal removal, as a major regulator of hormone secretion, and less of a consequence of pancreatic head removal [50].

The performance of Frey’s procedure in CP patients did not reduce the β-cell function or insulin resistance. In contrast, the surgery determined an α-cell dysfunction [51]. Similarly, there were no significant differences in endocrine outcome when comparing the Frey procedure with the Beger procedure for CP [52]. 

### 6.2. The Volume of Resection

Regarding the volume of the remaining pancreas after pancreaticoduodenectomy, Singh et al. [53] found that a percentage of <48.8% of residual pancreatic volume could be a significant risk factor for impaired glucose tolerance and progression to DM [53]. Similarly, an older study revealed that when the length of the removed pancreas was longer than 12 cm, the patients had a greater risk of developing DM [54].

In contrast, the amount of the resected pancreas and the procedure type were not found to be correlated with the prevalence of DM in patients undergoing pancreatic surgery for intrapapillary mucinous neoplasms (IPMNs) in the study by Leal et al. [55]. The degree of dysplasia was associated with increased risk of DM in this study.

### 6.3. Associated Risk Factors

A study performed by Schrader et al. analyzed the risk of developing DM in patients with partial pancreatectomy for CP. It showed that DM after partial pancreatectomy was predicted by pre-operative fasting glucose concentration, HbA1c level, and obesity, but not by reduced β-cell area. However, there was an inverse nonlinear relationship between pancreatic β-cell area and the fasting glucose or HbA1c level [56]. 

Another study by Menge et al. [57] investigated the β-cell capacity for recovery of glucose control after partial pancreatectomy in patients with chronic pancreatitis and benign pancreatic and extrapancreatic tumors. The results were in favor of an altered glucose homeostasis, including fasting and post-challenge glucose, insulin, and C-peptide concentrations immediately after surgery, but with a partial normalization during the follow-up of approximately 3 years. 

Surprisingly, there are cases where pancreatoduodenectomy may improve the glucose metabolism. In patients with pancreatic cancer, pancreatoduodenectomy may improve glycemic control through the decreased insulin resistance. A better effect is seen in patients with NOD than in those with long-standing DM. The possible explanation for this could be the discarding through resection of the paraneoplastic effect of pancreatic cancer on glucose metabolism and insulin resistance [58,59]. 

## 7. Pancreatic Adenocarcinoma

Pancreatic ductal adenocarcinoma (PDAC) is the most common pancreatic malignancy, with an overall 5-year survival rate barely reaching 10% [60]. The association between DM and pancreatic cancer has long been recognized; while long-standing diabetes has been reported to be associated with a risk increased by 1.5–2 times compared to that of non-diabetics, the risk of PDAC is highest in the first three years after diabetes diagnosis, up to 6–8 times higher than controls [61,62]. This strong temporal relationship between PDAC and diabetes duration has been shown in several studies, the risk being highest in the first year after diabetes diagnosis, then gradually decreasing for the next 1–4 years and reaching a plateau after 4 years [63]. With regard to the magnitude of the association, Pannala et al. showed that 85% of PDAC patients have altered fasting blood glucose levels (38% impaired fasting glucose and 47% in the range of diabetes), compared to 41% of controls (34% impaired fasting glucose and 7% diabetes) [59].

Beyond the dislocation of the parenchyma by the tumor itself, the mechanism of DM in pancreatic cancer is complex, involving a wide spectrum of cytokine and cell signaling as paraneoplastic phenomena, as well as the fall of β-cell identity and function and, finally, β-cell loss. The causality is also supported by the observation that NOD resolution occurs in >50% of patients after tumor resection, despite the reduced capacity for insulin secretion due to reduced β-cell mass [59,64,65].

### 7.1. Risk Factors

Among patients with PDAC, the greater incidence of DM was noted in those with higher body mass index and greater frequency of family history of DM [59]. 

Regarding the risk of PDAC among long-standing DM patients, several papers have focused on the role of hypoglycemic therapy in influencing this risk. Metformin has been shown to decrease PDAC risk by means of a liver kinase (LKB1-AMPK/liver kinase B-1–adenyl-monophosphate protein kinase), while incretin-mimetics comprising GLP-1 analogues and DPP (dipeptidyl peptidase) IV inhibitors have been theorized to increase risk through trophic effects on GLP-1 receptors, which are expressed not only on β-cells, but also on ductal and acinar cells [65]. Furthermore, insulin users are thought to have an increased PDAC risk, considering that hyperinsulinemia promotes cell proliferation [66].

On the other hand, NOD can often be harbinger of pancreatic cancer. The Enriching New-Onset Diabetes for Pancreatic Cancer Model (ENDPAC model), which was validated in several cohorts of NOD patients, has shown good accuracy in identifying patients at risk for pancreatic cancer. It revealed that diabetic patients who developed pancreatic cancer had a higher HbA1c level at diagnosis, as well as a more significant increase in HbA1c levels compared with the previous year, a lower body mass index at the moment of diagnosis, and a higher rate of tobacco and pancreatitis history [67,68,69]. Based on these observations, some authors have proposed some criteria to differentiate PDAC-associated DM from non-cancer-related progression of DM [70].

Furthermore, there is a significant impact of DM on PDAC prognosis with regard to the risk of perioperative complications, postoperative fistula, and mortality. A retrospective review by Lee, S. at al. [71] revealed that patients with PDAC and NOD have a worse oncological outcome and a high susceptibility to early recurrence after surgery [71].

### 7.2. Timing

**Figure 2:**
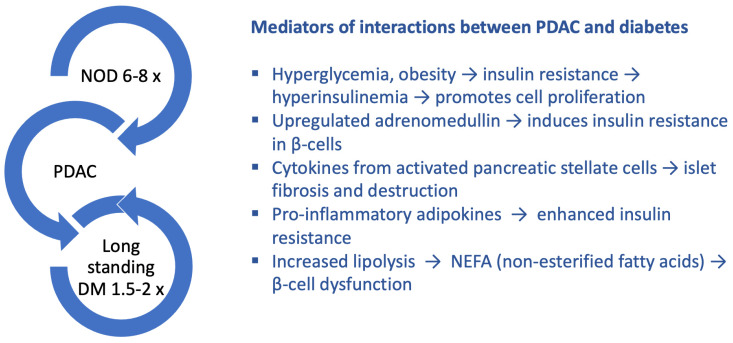
Mediators of interactions between PDAC and diabetes [61,78,79,80].

Longstanding T2DM increases the risk of pancreatic cancer by 1.5–2 times compared to that in the general population [61,72]. On the other hand, pancreatic cancer induces NOD, increasing the risk of DM diagnosis within 3 years to 6–8 fold in patients over the age of 50 years old with common risk factors for DM [73] (Figure 2). There is a strong correlation between altered fasting blood glucose and tumor volume, as hyperglycemia can precede PDAC diagnosis by 30–36 months [74]. These observations have fueled researchers to incorporate diabetes into screening strategies for PDAC when looking for enriching factors in NOD populations. 

A similar relationship with diabetes was also shown in neoplastic pancreatic cysts, which can constitute an alternative pathway to malignancy [75,76,77]. 

### 7.3. Mechanism

The mechanism of diabetes in PDAC has long been considered to be related to mechanical injury of the pancreatic parenchyma; however, in the light of more recent research, NOD is currently recognized to be caused by complex paraneoplastic phenomena [61] (Figure 2).

Histologically, DM related to pancreatic cancer has a unique pattern, which is different from that of T2DM and is characterized by the absence of amyloid aggregates and relatively preserved islet architecture. T2DM is morphologically and pathophysiologically characterized by amyloid deposition and a progressive decrease in β-cell mass, which is attributed to increased β-cell apoptosis and loss of β-cell potency and transcriptional identity. The deficit of β-cell mass in T2DM determines an increased α-cell to β-cell ratio, while in pancreatic cancer patients, both α-cell and β-cell area reductions are noted, with reduced islet size and islet density [73]. 

A hypothesis for the modifications seen in pancreatic-cancer-induced DM could be a paraneoplastic response due to metabolic changes. This idea is sustained by the evident correlation between the degree of hyperglycemia and the tumor size [74]. A pivotal role in tumor onset and progression is thought to be represented by carbonyl stress by means of hyperglycemia and dyslipidemia; in mouse models, blocking of reactive carbonyl species (RCS) and advanced glycation end-products (AGEs) prevented the progression of pancreatic intraepithelial neoplasia (PanIN) to invasive cancer [62].

Another hypothesis of β-cell failure in pancreatic cancer is the response to local and systemic cancer-induced inflammatory changes [81]. 

Finally, a mechanism could be the genetic alterations seen in pancreatic cancer, including in the genes regulating β-cell embryonic development [73].

As discussed for chronic pancreatitis, β-cell dedifferentiation was demonstrated to be a mechanism in the pathogenesis of pancreatic-cancer-associated DM, too, via loss of β-cell identity, a switch to a precursor status, and the failure of its secretory potency. β-cell dedifferentiation is thought to be induced by local and systemic paraneoplastic inflammation, and importantly, it precedes hyperglycemia [82]. The role of inflammation in the progression to DM in patients with pancreatic cancer is sustained by Gao et al. [83] in a study that shows higher levels of inflammatory markers in the serum of patients with pancreatic cancer and DM compared with those without DM and a decrease in these markers after tumor removal.

An interesting hypothesis for β-cell dysfunction proposed by Javeed et al. is that, in pancreatic cancer, there are extracellular vesicles containing adrenomedullin (AM) and CA19-9, which are responsible for impaired secretion of insulin. The exosomes are present in both portal and peripheral venous blood of pancreatic cancer patients. These exosomes enter β-cells through caveolin-mediated endocytosis and micropinocytosis, and an AM receptor interacts with its receptor on the β-cell, determining an upregulation of ER stress genes and an increase in reactive oxygen/nitrogen species, as well as perturbations in the unfolded protein response, leading to β-cell dysfunction and death [84]. Adrenomedullin mediates the inhibition of insulin secretion in β-cells in pancreatic cancer patients, and its level is higher in patients with pancreatic cancer than in patients with T2DM without pancreatic cancer [85].

Liu et al. investigated the mechanism of insulin resistance in skeletal muscle biopsies from pancreatic cancer patients with or without DM. The revealed mechanism was impaired glycogen synthesis and glycogen storage, and the removal of the tumor improved the insulin resistance and DM [86]. 

## 8. Autoimmune Pancreatitis

Autoimmune pancreatitis (AIP), a benign pancreatic disease that occurs alone or in association with other autoimmune conditions, is characterized by a chronic inflammation of the pancreas and is often accompanied by alterations in the glucose metabolism. 

### 8.1. Risk Factors—Steroid Treatment

DM was reported in 43–83% of AIP cases, while its improvement with steroid treatment was observed in 25–64% of cases [87]. The steroid treatment is a well-known therapeutical solution for AIP, but its role in glucose metabolism is not well understood. Steroid treatment improves the inflammation in AIP on the one hand, but on the other hand, it can induce insulin resistance and aggravate β-cell dysfunction. However, it seems that an early start with steroid treatment in AIP is the best option, as the recovery after insulin secretion that has been too severely damaged is extremely difficult. Hirano et al. [87] suggested that the indication of steroid treatment in AIP should be extended from symptomatic patients (as it is now) to all patients with at least impaired glucose tolerance in order to prevent exacerbations of endocrine dysfunction [87].

### 8.2. Timing

In a study including patients with coexisting AIP and DM, 56.9% of patients developed DM concurrently with AIP, 34.3% had DM before the onset of AIP, and 8.8% developed DM after the start of steroid treatment [88]. 

### 8.3. Mechanism

In the same study, the mechanisms revealed by the authors in the onset of DM concurrently with AIP were impaired blood supply to islet cells and damaged function of the islets because of inflammation and fibrosis [88]. The same hypothesis was mentioned in a Japanese study, in which rapid inflammation and fibrosis induced a reduction in blood flow, producing islet cell ischemia [89].

## 9. Conclusions

Alterations in glucose metabolism and evolution into DM are common complications of pancreatic exocrine diseases. As DM is one of the most common diseases worldwide, with serious systemic complications and a burden on healthcare systems, it is important to differentiate between preexisting diabetes and NOD in order to have a clear image of the probable evolution and therapeutical demands. This explains why screening for glucose metabolism impairment and DM has to be routine after flares of pancreatic exocrine diseases or in established diseases of the exocrine pancreas, not only at the moment of diagnosis, but also in the long-term follow-up.

## Figures and Tables

**Table 1:** Hormones secreted by the islet of Langerhans cells and the effects of their loss on glucose homeostasis [5,6].

Cell Type	Hormone Released	Action of the Hormone	Effect of Hormone Loss
α-cell	glucagon	- liver glycogenolysis and gluconeogenesis - paracrine action: promotes β-cells growth and survival by producing GLP-1 locally - capacity to transdifferentiate into β-cells	- severe hypoglycemia - low response to hyperglycemia - development of diabetes with reduced risk of ketoacidosis
β-cell	insulin	- hypoglycemia - stimulates hepatic glycogenesis and glycolysis - reduces hepatic gluconeogenesis and glycogenolysis - conversion of excess glucose into fatty acids and precursor triglyceride - transportation of intracellular glucose to insulin-dependent tissues, such as liver, muscle, and adipose tissue.	- hyperglycemia - increased hepatic gluconeogenesis, increased glycogenolysis
δ-cell	somatostatin	- inhibition of both insulin and glucagon release	- increases the risk of hypoglycemia
PP-cell	pancreatic polypeptide	- increases the hepatic insulin sensitivity	- induces hepatic insulin resistance

## Data Availability

Not applicable.
